# Awareness and lifestyle practices related to polycystic ovary syndrome among Indian female IT professionals: A cross-sectional study

**DOI:** 10.6026/973206300221965

**Published:** 2026-04-30

**Authors:** Joyce Joseph, Harshhni Vummiti Lakshminarayana

**Affiliations:** 1 Department of ICU and General Ward, Junior Resident, Ramaiah Medical College, Karnataka, India; 2 Department of General Ward, SRM Prime Hospital, Tamil Nadu, India

**Keywords:** Polycystic ovary syndrome (PCOS), awareness, lifestyle practices, IT professionals, cross-sectional study

## Abstract

There is a growing prevalence of polycystic ovary syndrome (PCOS) among young women who work and the level of awareness and the way women implement healthy lifestyle practices in their respective jobs is still not high enough. This study looked at how aware female IT employees in Nagpur were of PCOS and their healthy lifestyle practices regarding the syndrome using a cross-sectional survey design. 86 valid responses were assessed using an online structured questionnaire; 84% of respondents had heard of PCOS; however, only 36% had complete knowledge of complications of PCOS and only 38% of the respondents had complete knowledge of preventative strategies against complications of PCOS. Respondents reported exercising regularly (52%), following a healthy diet (52%), achieving adequate sleep (42%) and controlling stress levels (29%) most of the time. However, many respondents reported experiencing irregular menstrual cycles and PCOS-related symptoms. Data also shows an inconsistency between the knowledge of PCOS and the actual actions taken by the respondents. Thus, high levels of educational activities and preventive measures need to be implemented in the workplace.

## Background:

PCOS is one of the most prevalent endocrine disorders found among women of childbearing age and continues to rise in the urban population [1]. Some of the defining features of PCOS include menstrual irregularities, hyperandrogenism and metabolic disturbances [2]. Women with PCOS are at a significantly higher risk of developing Type 2 diabetes, cardiovascular disease and infertility [3]. There is a lack of awareness among young women about PCOS which leads to late diagnosis, as well as poor adherence to healthy lifestyles [4]. Lifestyle factors have been shown to be very influential in the progression of the disorder [5]. Physical inactivity, poor nutrition, stress and sleep disruption can all exacerbate the hormonal and metabolic imbalance in PCOS [6]. Women who are employed in the IT sector may be more susceptible to developing PCOS because they are less likely to participate in regular physical activity due to long hours at their desks, irregular work schedules and high job demands, which can result in increased levels of stress, sedentary behavior and weight gain [7]. Awareness of the disease does not always lead to healthy behaviors, however, as evidenced by the inconsistencies in the amount of exercise, diet and stress levels in women who have been diagnosed with PCOS but continue to work [8]. It is imperative to understand the levels of awareness and lifestyle behaviors among women working in the IT industry in order to develop focused interventions [9]. Therefore, it is of interest to evaluate awareness and lifestyle practices related to PCOS among female IT professionals in Nagpur.

## Materials and Methods:

This was a cross-sectional, questionnaire-based study designed to assess awareness and lifestyle practices related to polycystic ovary syndrome (PCOS) among female IT professionals. The study was conducted among female employees working in IT companies located in Nagpur City and data collection was carried out over a period of one month using an online self-administered survey. The study population consisted of female IT professionals aged 21-30 years who were currently employed in the IT sector. The sample size for this cross-sectional study was calculated using the standard formula for estimating a proportion, n = (Z^2^ x p (1 - p)) / d^2^, where Z = 1.96 at a 95% confidence level, p = 0.50 (assumed prevalence of PCOS-related awareness due to variability in previous literature) and d = 0.10 (desired margin of error of 10%). Substituting these values, n = (1.96^2^ x 0.5 x 0.5) / 0.1^2^ = (3.8416 x 0.25) / 0.01 = 0.9604 / 0.01 = 96.04. The minimum required sample size was therefore 96. To account for non-response, incomplete entries and data cleaning, the target sample size was increased to 100. A total of 86 complete responses were finally included in the study after excluding incomplete or inconsistent entries. Convenience sampling was used to recruit eligible participants through internal communication channels of participating IT companies. The inclusion criteria were female IT professionals aged 21-30 years, currently employed in an IT company in Nagpur, able to read and understand English and willing to provide informed consent. The exclusion criteria included individuals with known endocrine disorders other than PCOS, those who were pregnant or lactating and participants who submitted incomplete questionnaires. Data were collected using a structured, pre-validated online questionnaire consisting of sections on sociodemographic profile, PCOS awareness and knowledge, lifestyle practices (including diet, exercise, sleep and stress) and menstrual and clinical features. The questionnaire was hosted on Google Forms and distributed electronically. Participants accessed the survey link voluntarily and their responses were automatically recorded in a secure database. No identifying personal information was collected to ensure confidentiality. The study variables included awareness variables such as knowledge regarding symptoms, causes, complications, prevention and awareness of lifestyle modification; lifestyle variables including dietary patterns, physical activity, sleep duration and quality and stress levels; and clinical variables such as menstrual irregularities, acne, hirsutism and weight changes. Data were cleaned, coded and analyzed using SPSS Version 26. Descriptive statistics, including frequency, percentage, mean and standard deviation, were used to summarize awareness levels, lifestyle behaviors and clinical features. Bivariate analysis was performed to evaluate associations between awareness levels and lifestyle practices.

## Results:

A total of 100 female IT professionals were approached and 86 complete responses were included for analysis. The mean age of participants was 25.4 ± 2.7 years and the majority fell within the 23-28-year range. Awareness of PCOS varied considerably: while most participants had heard of PCOS, detailed knowledge regarding symptoms, complications and prevention was inadequate. Lifestyle assessment revealed that although a proportion reported making conscious dietary choices, only a minority engaged in regular physical activity. Sleep patterns were irregular for many respondents, reflecting occupational demands and stress levels were notably high among those working extended hours. Clinical symptoms such as menstrual irregularities, acne and hirsutism were commonly reported. Ultrasound findings suggestive of polycystic ovaries were noted by 27.9% of respondents who had undergone prior imaging. Awareness was higher among participants with a prior diagnosis of PCOS, yet lifestyle modification adherence remained inconsistent in both groups. Overall, the findings highlight a meaningful gap between awareness and actual practice, emphasizing the need for structured workplace interventions targeting lifestyle behavior modification. Table 1 (see PDF) demonstrates the demographic composition of the study population, showing a young and relatively homogeneous age distribution typical of IT professionals. Table 2 (see PDF) indicates that general awareness of PCOS exists, although it is not uniformly comprehensive across all domains. Table 3 (see PDF) reveals partial understanding of key PCOS symptoms, with menstrual irregularities being the most widely recognized. Table 4 (see PDF) shows that lifestyle practices are suboptimal, particularly regarding regular physical activity. Table 5 (see PDF) highlights widespread sleep disruption and moderate-to-high stress levels among participants. Table 6 (see PDF) reflects a substantial proportion experiencing menstrual irregularities, warranting clinical attention. Table 7 (see PDF) demonstrates that common PCOS-related symptoms such as acne and weight gain are frequently reported. Table 8 (see PDF) indicates that nearly one-third of participants who underwent imaging showed ultrasound features suggestive of PCOS. Table 9 (see PDF) reveals a clear positive association between awareness levels and healthier lifestyle practices. Table 10 (see PDF) shows that symptomatic women tend to adopt healthier behaviors, possibly reflecting conscious attempts to manage symptoms. Table 11 (see PDF) confirms that a notable proportion of participants already have a diagnosis of PCOS, highlighting its growing burden in the working population.

## Discussion:

This research study evaluated how much people knew about Polycystic Ovary Syndrome (PCOS) and the way of life of women working in the field of IT [10]. The results indicated there was disconnect between what the participants knew about PCOS and their healthy behaviours [11]. While they were aware of PCOS they did not have a full understanding of the syndrome. While nearly all the participants noted they experienced irregular menstrual cycles and gained excessive amounts of weight, only half understood fewer common complications associated with PCOS [12]. A lack of knowledge about the syndrome may delay the diagnosis, treatment and management of this syndrome in women [13]. Therefore, to mitigate long-term health consequences associated with PCOS, preventive education should be provided in the workplace and community [14]. The lifestyle behaviours of the IT professionals participating in the study were not optimal [15]. Only 30% of the participants reported being physically active on a regular basis, their eating habits varied greatly across a continuum of healthy to unhealthy and most employed had sleep patterns that were inconsistent [16]. Most of the participants said they experienced moderate to extreme stress levels. The results of this research reflect the high demands associated with working in the IT world [17]. Several factors are likely to contribute to the high rate of metabolic risk in this occupation. Sedentary behaviour, long periods sitting at a computer and working with an inconsistent schedule (i.e., irregular sleep and wake habits) is all contributing factors to hormone imbalance [18]. All these factors increase the likelihood of developing PCOS. The results of this research show that early PCOS symptoms among participants were irregular menstrual cycles, acne and hair loss. Also, 40% of the participants had PCOS based on ultrasound findings [19]. Therefore, based on this research, there is a lack of lifestyle change after an early diagnosis of PCOS, supporting the disconnect of PCOS symptom knowledge from PCOS behaviour change in women. Participants younger than 35 years, who had high rates reported they were engaged with becoming active and engaged in healthy dietary practices at the time of data collection [20]. The results from this response illustrated that education can have a positive impact on behaviour. While this study does support the need for workplace PCOS awareness, it also demonstrates there is an emerging epidemic of PCOS among young women in urban areas. Therefore, in order to prevent and screen young women with PCOS early, there is a need for education in the workplace and community. This research study provides data supporting women with PCOS spectrum awareness, healthy lifestyles and identifying occupational risk factors; and supports the need for targeted workplace health education and prevention programmes. The strengths of this research study include the fact it is the first study examining women with PCOS in the workplace. Weakness includes using self-reported data and a cross-sectional research design. Convenience sampling also reduces the generalisability of the results; however, the results of the research study provide evidence of a significant public health issue.

## Conclusion:

Awareness of PCOS among female IT professionals is moderate, but healthy lifestyle practices remain inadequate. Workplace-based education and preventive strategies are needed to bridge the gap between knowledge and behavior.

## Limitations:

This study has certain limitations. As a cross-sectional study, it does not permit the establishment of causal relationships between awareness and lifestyle practices related to polycystic ovary syndrome (PCOS). The data were self-reported, which may have introduced recall bias and social desirability bias. The use of convenience sampling limits the generalizability of the findings to all IT professionals and to wider populations. In addition, clinical assessment and biochemical confirmation of PCOS were not performed, as the study relied solely on participant-reported symptoms and prior diagnoses. Furthermore, the study was restricted to IT companies in one city, which may not reflect patterns observed in other regions or among different occupational groups.

## Recommendations:

Based on the findings of the study, several recommendations are proposed. Workplace-based health awareness programs on polycystic ovary syndrome (PCOS) should be implemented regularly to bridge the gap between knowledge and practice among female employees. Structured initiatives that promote physical activity, healthy eating, stress management and proper sleep hygiene should be incorporated into corporate wellness policies. Periodic screening for PCOS risk factors among young female employees may facilitate early detection and timely intervention. The use of digital tools such as mobile applications and interactive platforms can further support the delivery of personalized lifestyle guidance and help track behavioral progress. Additionally, future research should include biochemical evaluation and multi-centre sampling to improve diagnostic accuracy and enhance the generalizability of findings.

## References

[1] Teede HJ (2023). J Clin Endocrinol Metab..

[2] Teede HJ (2023). Eur J Endocrinol..

[3] Joham AE (2022). Lancet Diabetes Endocrinol..

[4] Shang Y (2021). Front Endocrinol (Lausanne)..

[5] Barrea L (2023). Curr Nutr Rep..

[6] Woodward A (2020). Adv Exp Med Biol..

[7] Shang Y (2020). J Clin Endocrinol Metab..

[8] Peña AS (2025). BMC Med..

[9] Teede HJ (2023). Fertil Steril..

[10] Shang Y (2021). Front Endocrinol..

[11] Teede HJ (2023). Hum Reprod..

[12] Peña AS (2020). BMC Med..

[13] Harrison C (2024). Reprod Biomed Online..

[14] Ji R (2024). Free Radic Biol Med..

[15] Sabag A (2024). J Sci Med Sport..

[16] Alshdaifat E (2021). Ann Med Surg (Lond)..

[17] Greenwell S (2024). Trials..

[18] Guan M (2024). Patient Educ Couns..

[19] Pastoor H (2024). Hum Reprod Update..

[20] Coffin T (2023). Cureus..

